# The impact of measuring split kidney function on post-donation kidney function: A retrospective cohort study

**DOI:** 10.1371/journal.pone.0253609

**Published:** 2021-07-02

**Authors:** Kelly C. Harper, Jean-Paul Salameh, Natasha Akhlaq, Matthew D. F. McInnes, Victoria Ivankovic, Mahdi H. Beydoun, Edward G. Clark, Wanzhen Zeng, Brian D. M. Blew, Kevin D. Burns, Manish M. Sood, Ann Bugeja

**Affiliations:** 1 Department of Radiology, The Ottawa Hospital, University of Ottawa, Ottawa, Ontario, Canada; 2 Ottawa Hospital Research Institute Clinical Epidemiology Program, Ottawa, Ontario, Canada; 3 Faculty of Medicine, University of Ottawa, Ottawa, Ontario, Canada; 4 Division of Nephrology, Department of Medicine, Kidney Research Centre, Ottawa Hospital Research Institute, University of Ottawa, Ottawa, Ontario, Canada; 5 Division of Nuclear Medicine, Department of Medicine, University of Ottawa, The Ottawa Hospital, Ottawa, Ontario, Canada; 6 Division of Urology, Department of Surgery, The Ottawa Hospital, University of Ottawa, Ottawa, Ontario, Canada; Imperial College Healthcare NHS Trust, UNITED KINGDOM

## Abstract

**Background:**

Studies have reported agreement between computed tomography (CT) and renography for the determination of split kidney function. However, their correlation with post-donation kidney function remains unclear. We compared CT measurements with renography in assessment of split kidney function (SKF) and their correlations with post-donation kidney function.

**Methods:**

A single-centre, retrospective cohort study of 248 donors from January 1, 2009-July 31, 2019 were assessed. Pearson correlations were used to assess post-donation kidney function with renography and CT-based measurements. Furthermore, we examined high risk groups with SKF difference greater than 10% on renography and donors with post-donation eGFR less than 60 mL/min/1.73m^2^.

**Results:**

62% of donors were women with a mean (standard deviation) pre-donation eGFR 99 (20) and post-donation eGFR 67 (22) mL/min/1.73m^2^ at 31 months of follow-up. Post-donation kidney function was poorly correlated with both CT-based measurements and renography, including the subgroup of donors with post-donation eGFR less than 60 mL/min/1.73m^2^ (r less than 0.4 for all). There was agreement between CT-based measurements and renography for SKF determination (Bland-Altman agreement [bias, 95% limits of agreement] for renography vs: CT volume, 0.76%, -7.60–9.15%; modified ellipsoid,1.01%, -8.38–10.42%; CC dimension, 0.44%, -7.06–7.94); however, CT missed SKF greater than 10% found by renography in 20 out 26 (77%) of donors.

**Conclusions:**

In a single centre study of 248 living donors, we found no correlation between CT or renography and post-donation eGFR. Further research is needed to determine optimal ways to predict remaining kidney function after donation.

## Introduction

Living donor kidney transplantation provides better patient and graft survival for patients with kidney failure as compared with deceased donor kidney transplantation [1,2]. However, the number of living kidney donor transplants has remained unchanged since 2008 while the need has increased in Canada, although the number of living kidney transplants has increased in other countries [3–5]. The evaluation process required for living kidney donation is costly and time-consuming, requiring extensive testing and multiple healthcare visits. Kidney donors have highlighted simplification of the donor evaluation process as a priority. Doing so may translate into increased numbers of living kidney donor transplants, leading to investigation of redundant testing and the efficiency of the donor candidate workup [6].

Abdominal computerized tomography (CT) is performed as part of the living donor workup in potential donors to assess the vasculature, help with surgical planning and excludes those with incidental findings and anatomy unsuitable for transplantation. Split kidney function testing also helps guide the selection of the kidney for transplantation, with a preference to leave the donor with the higher functioning kidney. Size measurements of each kidney are performed with current recommendations suggesting that nuclear renography (renography) be done to determine the function of each kidney (i.e. split kidney function) only in patients with a difference in kidney length exceeding 1 cm on CT [7,8]. However, it is unclear if this method alone should be adopted by all individual donor programs given the lack of long term data on their ability to predict post-donation remaining kidney function [9–14]. This is of particular concern for donors with a clinically significant difference in split kidney function on renography that is missed on CT and donors with low eGFR after donation. Several Canadian institutions still perform renography in all potential donors, including those performing a one day evaluation of donor candidates [8,15]. Our institution is amongst this group, currently reexamining our practice, which therefore supplies us with a large, non-consecutive population and data pool from recent years.

The objectives of our study were to determine whether CT-based measurements and renography can predict post-donation kidney function. We further specifically examined high risk individuals with split kidney function of greater than 10% on renography. We hypothesized that CT-based measurements would be equivalent to renography-based assessment of split kidney function and predicting post-donation kidney function.

## Materials and methods

The study protocol was approved by The Ottawa Hospital Health Science Network Research Ethics Board (Protocol ID 20190489-01H) and is posted to Open Science Framework (https://osf.io/yngqb/). The study is reported according to the The Strengthening the Reporting of Observational Studies in Epidemiology (STROBE) statement (S1 Table in S1 File) [16]. Patient identifiers were used for data collection but then de-identified at the point of analysis. The patients’ medical records were accessed October 2019 to September 2020 and no treatment sought by the patients. The Ottawa Hospital Research Ethics Board waived the need for informed consent for this study. Data contain potentially deidentifying and sensitive patient information and are only available from the Ottawa Health Science Network Research Ethics Board (www.ohri.ca/ohsn-reb, Civic Box 675, 725 Parkdale Avenue, Ottawa, Ontario K1Y 4E9 613-798-5555 ext. 16719 Fax: 613-761-4311) for researchers who meet the criteria for access to confidential data.

We conducted a retrospective cohort study of all consecutive living kidney donors in the Living Kidney Donor program at The Ottawa Hospital, a tertiary care hospital, from January 1, 2009 to July 31, 2019. All donors in our Living Kidney Donor program undergo nuclear renography, in addition to CT. The donor’s demographic information, kidney donated (left versus right), pre- and post-donation serum creatinine, dates of measurement, and estimated glomerular filtration rate (eGFR) by Chronic Kidney Disease Epidemiology Collaboration (CKD-EPI) equation were collected from the electronic health record. Our centre requires 2 separate estimates of GFR before donation and does not manadate direct GFR measurement, as per Canadian guidelines [8]. All donors with appropriate imaging available, including imaging performed at other institutions, were included. Donors were excluded if CT and nuclear renogram were performed more than 3 months apart, if imaging results were unavailable, or if imaging was incompatible with the imaging picture archiving and communication system (PACS) *(McKesson Technology Systems*, *GA*, *USA)* or Hermes workstations (*Hermes Medical Solutions*, *Stockholm*, *Sweden*). Additionally, donors were excluded if they had incomplete charts, defined as no available serum creatinine, date of donation, or follow up visits.

### Nuclear renography and CT-based measurements

Each donor underwent retrospective measurement of nuclear renography. The region of interest of each kidney was manually drawn to generate time-activity curve from the images. The areas of time-activity curves determined the split function of each kidney at 1–3 minutes. Nuclear renography at our institution would have initially been performed with 259–370 MBq (7.0–10.0 mCi) of technetium-labeled diethylenetriaminepentacetate (Tc99m-DTPA) scan or technetium-labeled mercaptoacetyltriglycine (Tc99m-MAG3). Dynamic images of the posterior or anterior and posterior abdomen were performed with a two-headed ECAM scanner (*Siemens Medical Solutions*, *USA*) for 30 minutes.

Three retrospective CT-based measurements were obtained: i) two dimensional craniocaudal (CC) dimension ii) two dimensional modified ellipsoid volume and iii) three dimensional volume. Two-dimensional CT measurements were obtained on PACS software with manipulation of the imaging to provide an oblique orientation along the kidney’s true long axis. Modified ellipsoid volume was calculated volume = length x width x thickness x (pi/6) according to Soga et al. to allow volume inference from 2D measurements, requiring craniocaudal (length), laterolateral (width), and anteroposterior (thickness) measurements [17]. Three dimensional CT volumetry was performed with AquariusNET software *(TeraRecon*, *Foster-City*, *California*) using semi-automated contouring with exclusion of surrounding structures, including vasculature and collecting systems. CT measurements were obtained in the corticomedullary phase wherever available. If unavailable, arterial phase was preferentially used over non-contrast phase imaging to provide increased contrast resolution between adjacent tissues.

Two investigators (third year diagnostic radiology residents, KH and NA) performed all imaging measurements independently to determine inter-rater agreement. One investigator (KH) performed measurements twice for intra-rater agreement, with a period of at least one week between measurements. Investigators were blinded to prior measurements or any clinical outcomes.

### Measurement of split kidney function

Split kidney function was calculated as right minus left kidney (right−left) for all imaging modalities. Nuclear renography measurements are expressed as percentage function based on scintigraphic integral method inference. Split kidney function from CT-based measurements assumed the individual kidney contribution to overall kidney function was proportional to size and inferred by converting measurements to percentages based on the equation (right-left)/(right+left) x100%. A greater than 10% difference between right and left split kidney function was considered to be clinically significant [7,9,10].

### CT and renography-based prediction of remaining kidney function following kidney donation

Patients with at least 6 months post-donation data available, specifically CKD-EPI eGFR, were identified. Predicted post-donation eGFR was calculated as pre-donation eGFR x % split kidney function of the retained kidney for each modality. Correlation between predicted and observed post-donation eGFR was determined using Pearson’s correlation coefficient for all donors and the subgroup of donors with low eGFR (less than 60 mL/min/1.73m^2^). Correlation coefficients were interpreted as follows: <0.3 negligible, 0.3–0.5 low, 0.5–0.7 moderate, 0.7–0.9 high, and 0.9–1.0 very high correlation [18].

### Donors with split kidney function greater than 10% on nuclear renography

Donors with a split kidney function difference of greater than 10% on renography were identified. Their kidney donated and post-donation remaining kidney function at last follow-up was determined. Donors within this group were examined for having a split kidney function difference of greater than 10% on renography that was not found on any CT-based measurement.

### Agreement and reproducibility between CT and nuclear renography measurements

Bland-Altman analysis was used to evaluate agreement between each of the CT-based measurements of split kidney function and renography. Intra- and inter-rater agreement between CT and renography measurements was performed using intraclass correlation (ICC) analysis. Intra-rater agreement was calculated using the mean values for the reader (KH) with two scan measurements and inter-rater agreement was calculated using the first of the reader’s two measurements. ICC values were interpreted as follows: <0.5 poor, 0.5–0.75 moderate, 0.75–0.9 good, and >0.90 excellent reliability [19].

### Statistical analysis

Baseline characteristics and outcomes are presented as means (± standard deviation, SD) for continuous variables and frequency (percentage) for categorical variables. Statistical significance was defined as a two-sided p<0.05. All statistical analyses were performed in R (R Foundation for Statistical Computing, Vienna, Austria).

## Results

**Fig 1:**
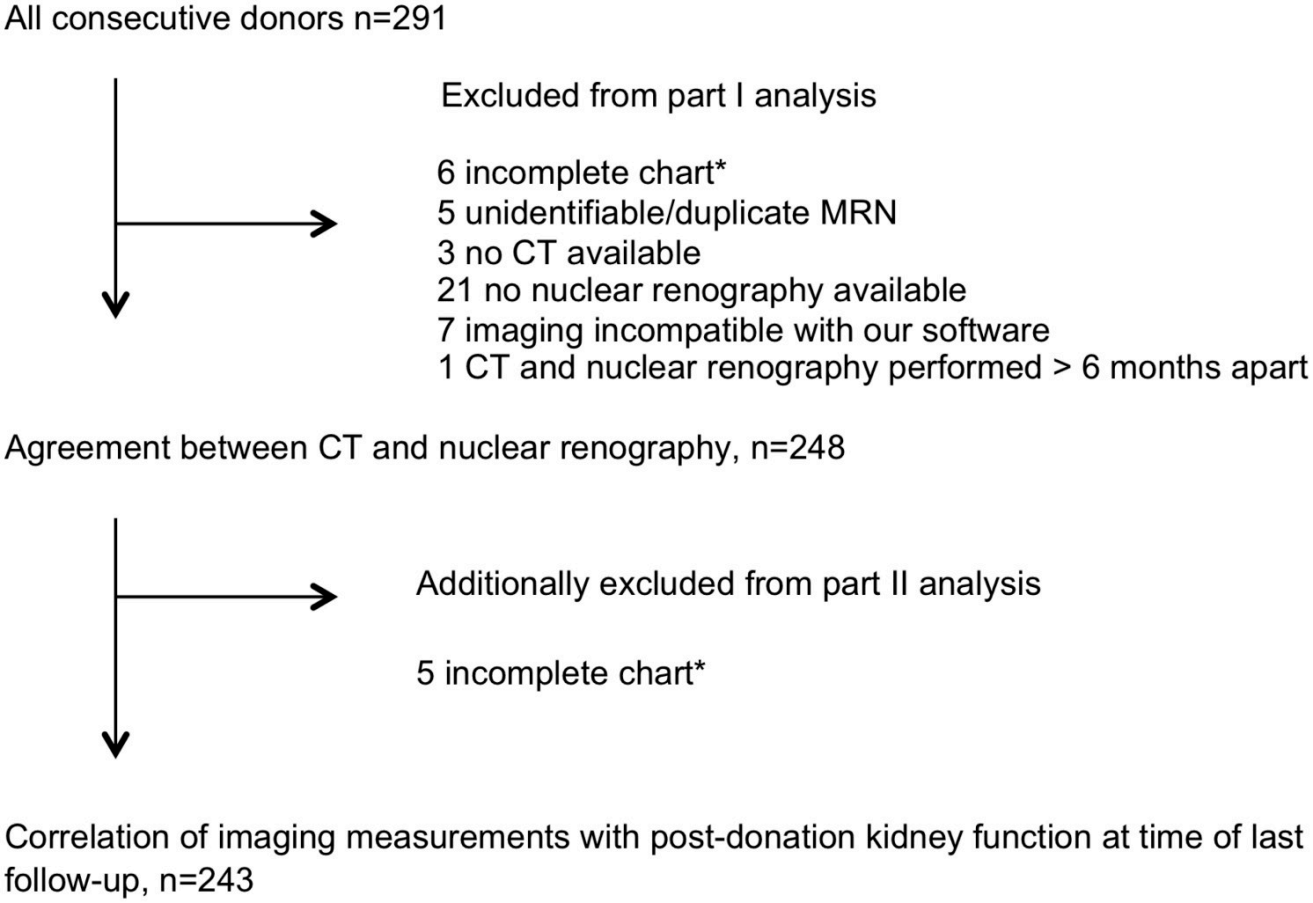
Study recruitment. *Incomplete defined as no available serum creatinine, date of donation, or follow up visits. CT, computed tomography.

Two hundred and ninety-one consecutive living kidney donors were identified. 248 donors had both CT and nuclear renography imaging available and 243 donors had post-donation eGFR available (Fig 1). 154 (62%) were female and 219 (88%) Caucasian. Mean (SD) age at donation was 48 (13) years and duration of follow-up post-donation was 31 (21) months (Table 1). Mean (SD) pre-donation was eGFR 99 (20) mL/min/1.73m^2^ and post-donation eGFR 67 (22) mL/min/1.73m^2^. The left kidney was donated in 222 donors (89%). 227 (93%) donors had a post-donation CKD-EPI eGFR available at a minimum 6 months after donation. 96 (40%) of donors had a post-donation eGFR of less than 60 mL/min/1.73m^2^.

**Table 1:** Baseline characteristics of live kidney donor study population, n = 248.

Age (years), mean (SD)	48 (13)
Female, n (%)	154 (62)
Body mass index, kg/m^2^, mean (SD)	28 (4)
Kidney donated, n (%)	
Right	21 (9)
Left	221 (89)
Unknown	6 (2)
Ethnicity, n (%)	
Caucasian	219 (88)
African American	11 (4)
Aboriginal	4 (2)
Other	14 (6)
Pre-donation eGFR (SD)	99 (20)
Post-donation eGFR (SD)	67 (22)
Time to most recent post donation eGFR (months), mean (SD)	31 (21)

SD, standard deviation. eGFR reported as mL/min/1.73m^2^.

### CT and renography-based prediction of remaining kidney function following kidney donation

**Fig 2:**
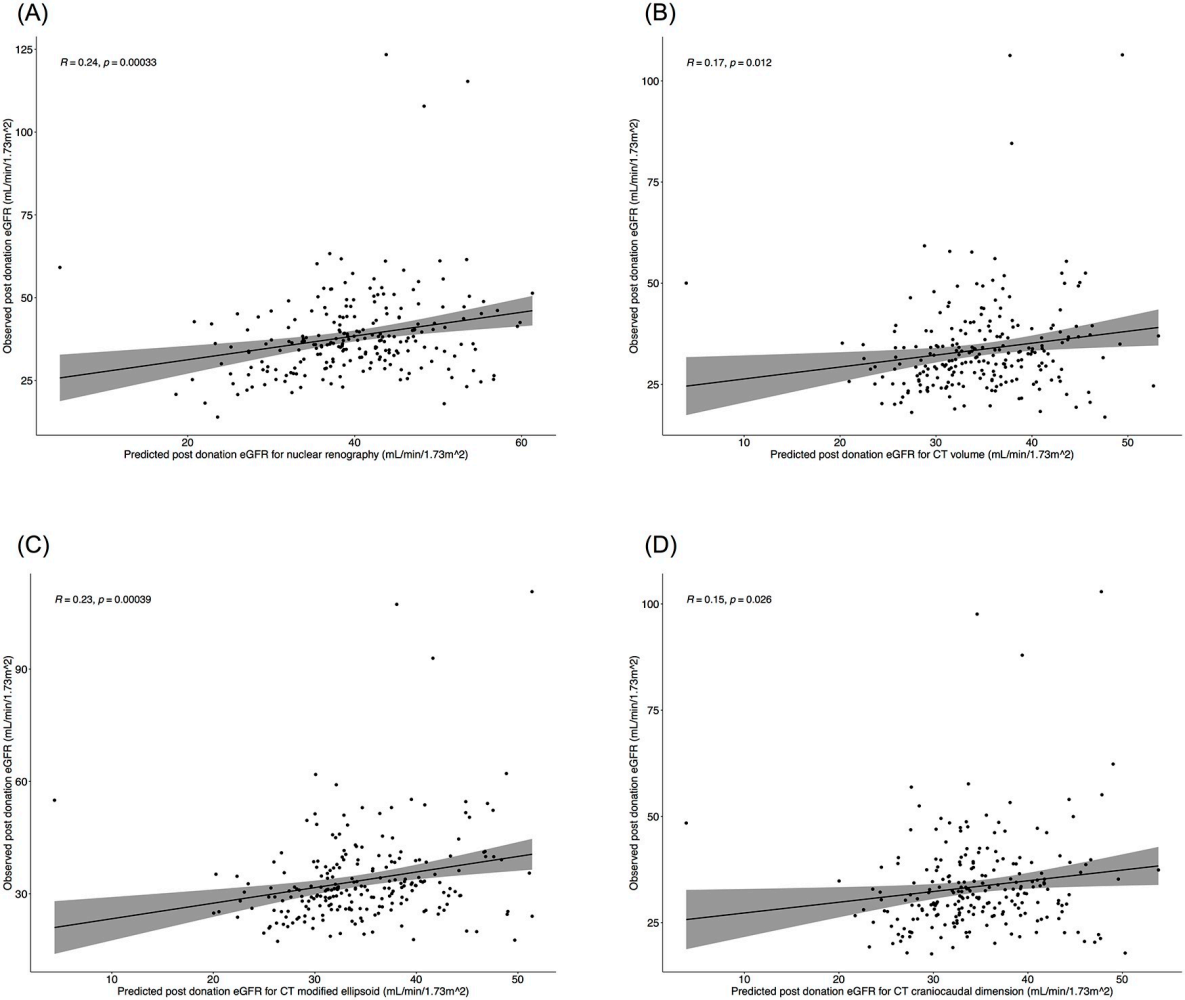
A–D. Pearson’s correlation of predicted and observed eGFR by modality. Predicted eGFR of A) nuclear renography B) CT volume C) CT modified ellipsoid volume and D) CT craniocaudal dimension correlated with observed post-donation eGFR in donors with most recent eGFR available at a minimum of 6 months, n = 227.

**Fig 3:**
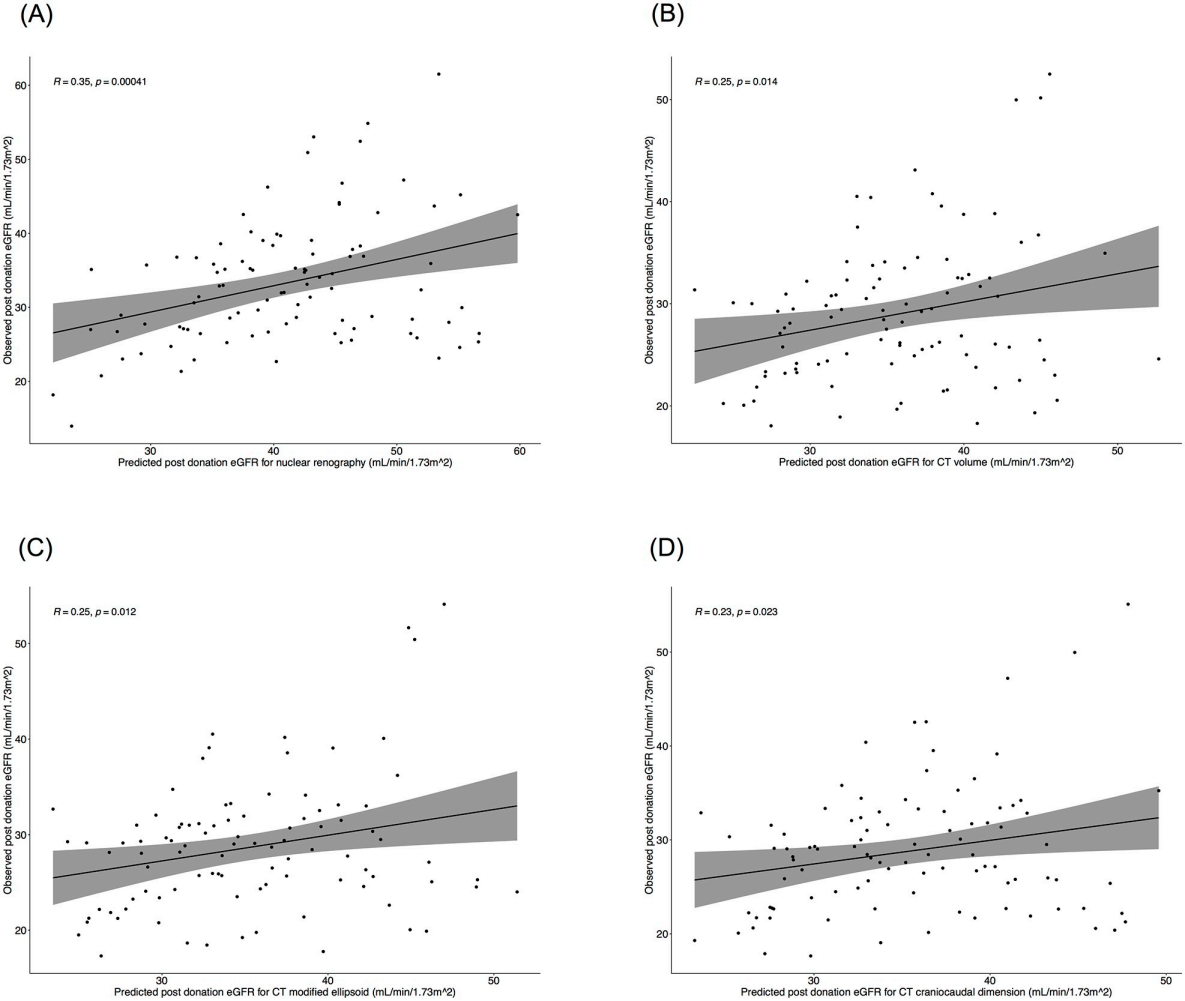
A–D. Pearson’s correlation of predicted and observed eGFR by modality in patients with low eGFR. Predicted eGFR of A) nuclear renography B) CT volume C) CT modified ellipsoid volume and D) CT craniocaudal dimension correlated with observed post-donation eGFR in donors with most recent eGFR of less than 60 mL/min/1.73m^2^, n = 96.

Correlation of predicted eGFR with observed post-donation eGFR in all four imaging measurements (nuclear renography, CT volume, modified ellipsoid, CC dimension) are depicted in Fig 2A-2D for all donors with a minimum 6 months of follow up, n = 227 (93%). There was negligible correlation for all 3 CT-based measurements and low correlation on renography. Among the subgroup of donors with most recent eGFR less than 60 mL/min/1.73m^2^, n = 96 (40%) (Fig 3A-3D), there was negligible correlation for all 3 CT-based measurements and low correlation for renography.

### Donors with split kidney function greater than 10% on nuclear renography

Twenty six (11%) donors had greater than 10% difference in split kidney function based on nuclear renography which was missed by at least 1 CT-based measurement in 20 (77%) donors (Table 2). Among these 26 donors, 9 (35%) donated their higher functioning kidney (Table 3). 6 (23%) of the 26 donors had a post-donation eGFR of less than 60 ml/min/1.73m^2^ at a mean follow-up from donation to most recent post-donation eGFR measurement of 37 ± 22 months (Table 3). Only 1 of the 6 donors who donated their higher functioning kidney had an eGFR of less than 60 mL/min/1.73m^2^ at 53 months post-donation. Among the 9 (35%) donors who donated their higher functioning kidney, the mean pre-donation eGFR was 101 mL/min/1.73m^2^ and post-donation eGFR was 71 mL/min/1.73m^2^ while in the 17 donors who donated their lower functioning kidney the mean pre donation was 98 mL/min/1.73m^2^ and mean post-donation was 72 mL/min/1.73m^2^. Overall, the mean eGFR decline for those who donated their higher functioning kidney was 30 mL/min/1.73m^2^ and for those who donated their lower functioning kidney was 26 mL/min/1.73m^2^.

**Table 2:** Agreement between modalities when difference in split kidney function is greater than/equal to or less than/equal to 10%, n = 248.

	Renography ≤±10%	Renography >±10%
CT Volume ≤±10%, n (%)	206 (83)	24 (10)
CT Volume >±10%, n (%)	16 (6)	2 (1)
CT Modified ellipsoid ≤±10%, n (%)	186 (75)	20 (8)
CT Modified ellipsoid >±10%, n (%)	36 (15)	6 (2)
CT CC ≤±10%, n (%)	220 (89)	26 (10)
CT CC >±10%, n (%)	2 (1)	0

CC = craniocaudal dimension. Donors may be counted more than once because the difference in split kidney function may have varied between the 3 CT measurements.

**Table 3:** Characteristics for donors with >10% SKF difference on renography, n = 26.

Patient	Gender	Age at donation	Kidney donated	Renography SKF*	Higher functioning kidney donated?	Pre donation eGFR	Post donation eGFR	% change	Months post donation
1	M	63	L	-11	Y	79	53	-33	53
2^+^	F	60	L	-11	Y	89	63	-29	43
3^+^	F	50	L	11	N	76	55	-28	1
4	F	49	L	11	N	108	79	-27	12
5^+^	M	25	L	-11	Y	93	69	-26	52
6^+^	F	49	R	-12	N	105	74	-30	37
7	M	35	L	12	N	103	75	-27	63
8^+^	F	68	L	12	N	94	88	-6	46
9^+^	F	33	L	-12	Y	86	65	-24	35
10^+^	F	63	L	13	N	89	63	-29	84
11^+^	F	62	L	13	N	93	49	-47	69
12^+^	F	55	L	13	N	89	62	-30	12
13^+^	F	38	L	-13	Y	102	72	-29	49
14^+^	M	25	L	-13	Y	114	84	-26	25
15^+^	M	41	L	-13	Y	109	62	-43	0
16	F	34	L	13	N	118	81	-31	24
17^+^	F	46	L	14	N	85	57	-33	42
18^+^	F	85	L	-14	Y	127	99	-22	12
19^+^	F	40	R	-14	N	88	68	-23	53
20	M	56	L	15	N	95	56	-41	12
21^+^	M	24	L	15	N	126	98	-22	50
22	F	57	L	17	N	94	81	-14	47
23^+^	F	29	R	-17	N	124	96	-23	33
24^+^	F	65	L	18	N	91	81	-11	48
25^+^	F	46	R	-18	N	90	58	-36	6
26^+^	F	49	L	-20	Y	106	69	-35	44

SKF, split kidney function. M, male. F, female. L, left. R, right. Y, yes. N, no. eGFR reported in mL/min/1.73m^2^.

*Positive SKF indicates a higher functioning right kidney.

^+^Indicates a donor with >10% SKF not identified on any CT measurement.

### Donors with post-donation eGFR <60 mL/min/1.73m^2^

96 of the 243 donors with an available post-donation eGFR (40%) had a post-donation eGFR of less than 60 mL/min/1.73m^2^ at last follow-up, with mean (SD) follow up of 28 (27) months. The mean (SD) pre-donation eGFR of this group was 90 (25) mL/min/1.73m^2^ and post was 51 (6) mL/min/1.73m^2^. 39 (41%) of these 96 patients donated their higher functioning kidney and their mean (SD) pre-donation eGFR was 89 (28) mL/min/1.73m^2^ and post-donation eGFR was 49 (7) mL/min/1.73m^2^. Split kidney function of greater than 10% identified on renography was only seen in 7 donors, 2 of whom were identified by 1 or more CT-based measurements.

### Agreement and reproducibility between CT and nuclear renography measurements

**Fig 4:**
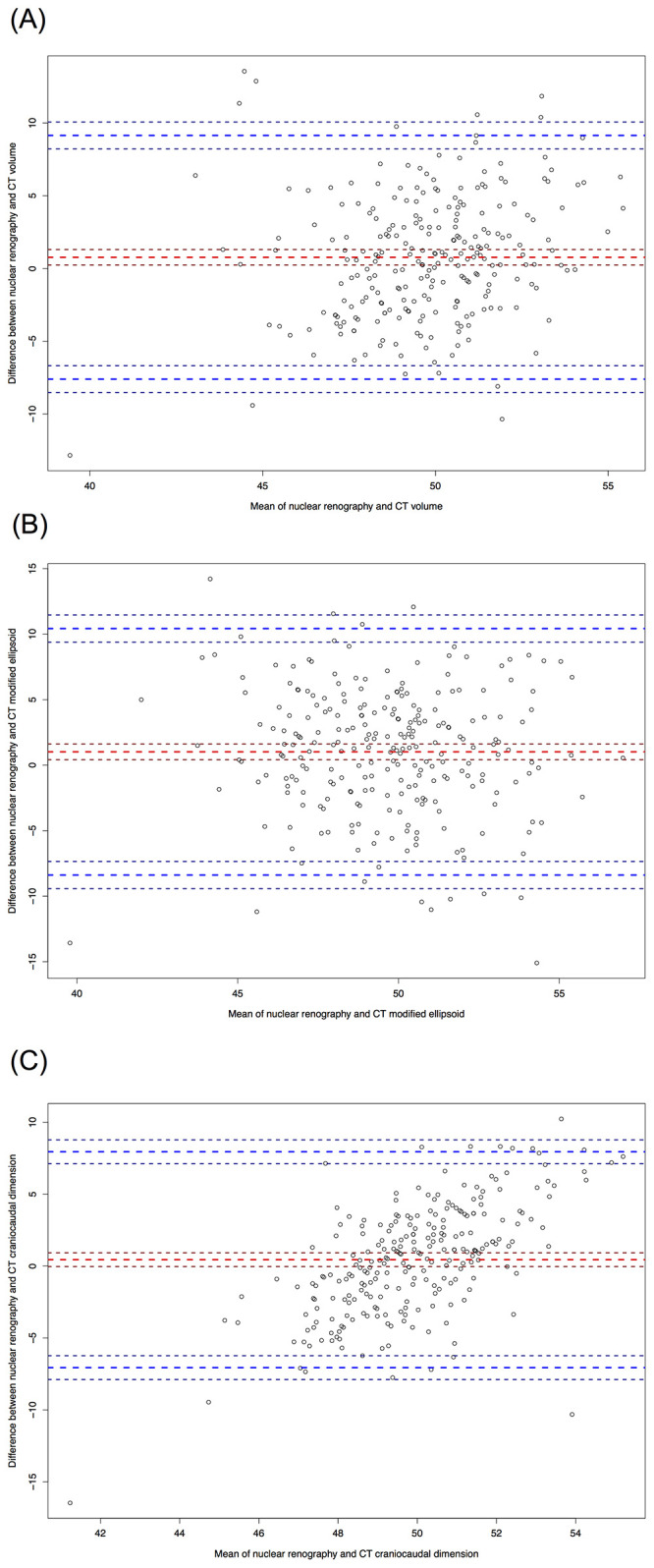
A–C. Bland Altman plots of agreement of split kidney function measured by nuclear renography versus CT based measurements. Nuclear renography based measurements versus A) CT volume with mean bias, (thick red perforated line) & 95% limits of agreement (thick blue perforated lines) of 0.76%, -7.60–9.15%, B) CT modified ellipsoid 1.01%, -8.38–10.42%, and C) CT craniocaudal dimension 0.44%, -7.06–7.94, n = 248. Thin perforated lines represent standard deviations.

Bland-Altman agreement between nuclear renography and CT measurements (mean bias, 95% limits of agreement) for renography vs: CT volume, 0.76%, -7.60–9.15%; modified ellipsoid, 1.01%, -8.38–10.42%; CC dimension, 0.44%, -7.06–7.94 (Fig 4A-4C). Intra-rater agreement was excellent for nuclear renography (ICC 0.92). Intra-rater agreement was moderate for CT-based measurements: volume, ICC 0.6; modified ellipsoid, ICC 0.57; and craniocaudal dimension, ICC 0.66. There was good inter-rater agreement for nuclear renography (ICC 0.86) and moderate agreement for CT-based measurements: volume, ICC 0.64; modified ellipsoid, ICC 0.64; and craniocaudal dimension, ICC 0.72 (Table 4).

**Table 4:** Intra- and Inter-rater agreement for nuclear renography and CT based measurements assessed by intra-class correlation coefficient (ICC), n = 248.

	Intra-rater agreement	Inter-rater agreement
Nuclear Renography	0.92 (0.90–0.94)	0.86 (0.73–0.92)
CT Volume	0.60 (0.49–0.69)	0.64 (0.54–0.72)
CT Modified Ellipsoid	0.57 (0.45–0.66)	0.64 (0.54–0.72)
CT CC Dimension	0.66 (0.59–0.73)	0.72 (0.64–0.78)

CC, craniocaudal dimension. CT, computed tomography.

## Discussion

In this large cohort of living kidney donors, neither CT-based measurement nor renography predicted kidney function at a mean of 31 months of post-donation follow-up. Furthermore, this was consistent among those deemed high clinical risk with a split kidney function greater than 10% on renography or eGFR< 60 mL/min post-donation. Lastly, despite a high level of CT and nuclear renography for SKF measures, a surprising CT missed a clinically significant split kidney function difference found by renography in 20 out 26 (77%) of donors.

Renography is routinely used in many donor programs due to concerns of missing a clinically significant difference in split kidney function with the ultimate concern of a donor having a low post-donation remaining kidney function of less than 60 mL/min/1.73 m^2^. In our study, neither CT-based measurements nor renography predicted post-donation eGFR in this clinically relevant subgroup. Most patients did not have a clinically significant difference in split kidney function by renography or CT-based measurements, but a small number of donors were found to have a difference identified on renography that CT would have missed. These clinically relevant outliers with a significant difference in split kidney function on renography not found on CT also create concern about post-donation remaining kidney function for clinicians and the potential for adverse outcomes in patients undergoing an altruistic, elective nephrectomy. Our study adds granular data outlining which kidney was donated and their post-donation kidney function with longer follow-up of 37 months for this group. Although post-donation kidney function appeared similar among donors with a clinically significant difference in split kidney function found on renography but not CT, this number is small and precluded further analysis around the ability of renography or CT to predict post-donation kidney function in this group of donors, an important question for future research. Furthermore, split kidney function was not the only determinant of kidney selection for donation as some donated their higher, rather than lower, functioning kidney.

Our study differs from other studies that found stronger prediction of post-donation eGFR [9–11]. The reason for this difference may relate to our longer follow up time while prior studies examined eGFR at only 6 to 12 months [9–11]. However, in keeping with previous studies, our Bland-Altman analysis confirmed that the majority of difference scores between renography and each of the 3 CT-based measurements were within the 95% confidence interval of the differences, indicating agreement between simple CT-based measurements and nuclear renography for the estimation of split kidney function similar to prior studies [9,10,14,17,20].

Strengths of our study include data derived from a large, non-selected donor cohort as all donor candidates had nuclear renography, and the performance of inter-rater agreement (having 2 independent assessors), which many studies did not examine for the consideration of reproducibility [9–11,13,14,21]. Studies that pre-select donor populations based on imaging results may be biased towards increased likelihood of finding a greater than 1 cm difference in kidney length and therefore discrepant split kidney function, whereas our study includes all consecutive donors since renography was performed for all donors in the program [14,17,22–24]. Limitations of our study include its retrospective nature, single-centre study design and lack of direct GFR measurement. The Kidney Disease: Improving Global Outcomes Guideline Evaluation and Care of Living Kidney Donors acknowledges that both measured GFR and eGFR are associated with error [7]. Furthermore, KDIGO recommends that post-donation eGFR be assessed by serum creatinine level since alternate methods are unavailable (cystatin C), impractical (24 hour urine collections for creatinine clearance), and expensive (nuclear measurement) for routine surveillance [7]. Because it is unclear whether one method outperforms another in predicting long term kidney function post-donation, we were reassured to use eGFR before and after donation to assess kidney function in relation to imaging. Given that eGFR increases by 1 ml/min/1.73 m^2^ per year on average until 5 years after donation, the lack of serial measurements of eGFR at various time points is also limitation of our study [25]. We did not collect data on comorbidities present before or after donation and our study is limited to a predominantly white population.

Moving forward with our institutional living kidney donor program, we will continue to use nuclear renography for the routine estimation of split kidney function, since there is a risk of missing a clinically significant difference in split kidney function by using CT alone. For the small number of donors with a clinically significant split kidney function difference on renography—most of whom would be missed by CT measurement estimates of split kidney function—there does not appear to be any difference in post-donation kidney function among those who donated their higher or lower functioning kidney but these numbers are small and may not be representative of a larger population. Although the use of CT-based measurements for the routine estimation of split kidney function will reduce the time, complexity, and cost associated with evaluation of living kidney donor candidates, our data does not support changing our approach to use CT-based measurements alone.

## Supporting information

S1 File STROBE statement. Checklist of items that should be included in reports of observational studies with appropriate page listings provided.
(PDF)
